# Three-Step Synthesis of a Redox-Responsive Blend of PEG–*block*–PLA and PLA and Application to the Nanoencapsulation of Retinol

**DOI:** 10.3390/polym12102350

**Published:** 2020-10-14

**Authors:** Louise Van Gheluwe, Eric Buchy, Igor Chourpa, Emilie Munnier

**Affiliations:** 1 EA 6295 Nanomédicaments et Nanosondes, Faculté de Pharmacie, Université de Tours, 31 Avenue Monge, 37 200 Tours, France; louise.vangheluwe@univ-tours.fr (L.V.G.); igor.chourpa@univ-tours.fr (I.C.); 2 Laboratoires Eriger, 39 Rue des Granges Galand, 37550 Saint-Avertin, France; eric.buchy@laboratoires-eriger.com

**Keywords:** redox responsive PEG-*block*-PLA, nanocarriers, disulfide bond, controlled release, retinol

## Abstract

Smart polymeric nanocarriers have been developed to deliver therapeutic agents directly to the intended site of action, with superior efficacy. Herein, a mixture of poly(lactide) (PLA) and redox-responsive poly(ethylene glycol)–*block*–poly(lactide) (PEG–*block*–PLA) containing a disulfide bond was synthesized in three steps. The nanoprecipitation method was used to prepare an aqueous suspension of polymeric nanocarriers with a hydrodynamic diameter close to 100 nm. Retinol, an anti-aging agent very common in cosmetics, was loaded into these smart nanocarriers as a model to measure their capacity to encapsulate and to protect a lipophilic active molecule. Retinol was encapsulated with a high efficiency with final loading close to 10% *w/w*. The stimuli-responsive behavior of these nanocarriers was demonstrated in vitro, in the presence of l-Glutathione, susceptible to break of disulfide bond. The toxicity was low on human keratinocytes in vitro and was mainly related to the active molecule. Those results show that it is not necessary to use 100% of smart copolymer in a nanosystem to obtain a triggered release of their content.

## 1. Introduction

Stimuli-responsive nanocarriers (NCs) have been designed to control the release of active molecules into the intended site of action. The contents of the nanoparticle is released only if a biological or a physical stimulus occurs. The stimulus has to be adapted to the application. For example, light-responsive, ultrasound-responsive, temperature-responsive, pH-responsive or redox-responsive nanosystems can be found in the literature [1].

Lately, a lot of efforts have been focused on the development of redox-responsive nanosystems. They could be of major interest in the treatment of cancer, as the reduction potential of cancer cells is higher than the reduction potential of healthy cells [2]. The release is then triggered in the tumor, minimizing the side effects. It is the observed case in certain skin cancers, such as melanoma [3,4]. Moreover, when skin is exposed to UV light, cells react first by activating antioxidant mechanisms: the level of glutathione (GSH) and its accompanying enzymes is dramatically increased to counteract the appearance of reactive oxygen species [5]. The increased GSH activity could favor the nanocarriers’ material cleavage and thus enhance the release of hydrophobic drugs [6,7].

Different NCs engineered with redox sensitive mechanisms for drug or gene delivery have been developed already like liposomes, polymer nanoparticles, micelles, or prodrug-based delivery systems [8,9]. The disulfide bond is the most used reduction-sensitive linker to prepare redox-responsive nanocarriers [10]. The disulfide bond can be localized in different places: it can make the link between two copolymers to form a stimulus-sensitive copolymer [11,12], it can make the link between the polymer and the drug to synthesize a redox-responsive conjugate [13], or it can be located in each repeating unit of a polymer [14,15]. In recent decades, significant efforts have been made in the development of stimuli-responsive polymers [16,17] to prepare efficient triggered release. Indeed, compared to conjugation, the synthesis of redox-responsive diblock or triblock copolymers is interesting as numerous active molecules can be encapsulated without developing a specific synthesis path for each one. Under the action of a stimulus, the copolymer disassembles into two or three parts, destabilizing the nanocarriers and leading to the release of the contents. Among the redox-responsive copolymers, the poly(ethylene glycol)–*block*–poly(lactide) (PEG–*block*–PLA) copolymer associated with a disulfide bond seems to still be the preferred iteration [6,7,18]. Indeed, both polymers PLA and PEG are approved by the pharmaceutical and cosmetic regulatory authorities. PLA is a synthetic linear aliphatic polyester that has proven its interest in the encapsulation of hydrophobic drugs [19,20]. Being biodegradable and biocompatible, it is used in various biomedical applications. PEG is a biocompatible polymer known for its hydrophilicity, flexibility, and non-toxicity. PEG confers to PEG–*block*–PLA-based nanocarriers a good stability in aqueous suspensions thanks to its hydrophilicity and sterical hindrance, and most of the time no adjunction of surfactant is necessary [21]. Moreover, PEG seems to play a positive role in the penetration of PLA-based nanosystems into the skin [22].

PEG–*block*–PLA-based nanocarriers have already proven their efficacy in the encapsulation of siRNA, active pharmaceutical or cosmetic ingredients [23,24,25], among which is retinol. Retinol is a low molecular weight anti-ageing agent very common in cosmetics [26]. It is highly sensitive to water, heat, oxygen and light [27,28,29,30]. Many authors work on its encapsulation to protect its integrity and increase its penetration into the skin [31,32,33,34]. NCs made of PEG–*block*–PLA copolymer showed very interesting performances for retinol delivery to the skin [34,35]. Retinol loaded PEG–*block*–PLA-based NCs displayed a significantly higher absorption into skin compared to PEG–*block*–PCL NCs, surfactant micelles, or oil solution [34]. As a powerful antioxidant molecule, it could benefit from an encapsulation in a redox-sensitive PEG–*block*–PLA to confer a triggered release in case of skin photooxidation. 

Unfortunately, the syntheses of redox-responsive PEG–*block*–PLA described in the literature are multi-stage, with drastic anhydrous conditions, and time-consuming purification steps requiring specific equipment [7,18,36,37]. These constraints limit the potential interest of such delivery systems. Moreover, PEG–*block*–PLA nanocarriers are usually nanomicelles showing low physical stability in suspension and showing a low resistance to dilution, which is not compatible with the introduction into skin dedicated products like creams or gels. The stability can be increased by mixing it with another polymer that will reinforce the hydrophobic core of the nanocarriers, for example PEG–*block*–PLA mixed with PLA. In this case, both polymers are usually synthesized independently [22,38].

The objective of the present study was to prepare redox-responsive nanocarriers made of a blend of PLA and a PEG–*block*–PLA copolymer linked with a disulfide bond. Such nanocarriers should be stable in aqueous suspension but release their contents rapidly in skin undergoing oxidative stress. In order to attain this goal, the steps were (1) to prepare in as few steps as possible a redox-responsive blend of PEG–*block*–PLA and PLA; (2) to formulate retinol nanocarriers via a simple and efficient protocol. For the synthesis of the redox-responsive PEG–*block*–PLA copolymers blended with PLA, a new strategy in only three steps was developed. At the end, a redox-responsive blend of PEG–*block*–PLA and PLA was obtained and used directly, without further purification. It was chosen to work with short PLA chains in order to facilitate the release of retinol, which could be long in long-chain PLA nanocarriers despite the redox-responsive link, because of an increased affinity of retinol for the polymer [39]. To formulate the nanocarriers encapsulating retinol, the nanoprecipitation method was chosen because it is well known, fast and easy to scale up. This method is the simplest preparation method of nanocarriers as it takes place in only one step, with no need of surfactants or chlorinated solvents [40]. It leads to a narrowly dispersed particle size. The impact of GSH levels on redox-responsive release was investigated in vitro. Finally, cytotoxicity of the nanocarriers on human keratinocytes was evaluated to ensure that they could be safely used in dermatological or cosmetic applications.

## 2. Materials and Methods

### 2.1. Chemicals

2-mercaptoethanol, 2,2′-dithiobis(5-nitropyridine), anhydrous acetic acid, methanol, O-[2-(3-mercaptopropionylamino)ethyl]-O′-methylpolyethylene glycol (PEG-thiol, 5 kDa), 3,6-dimethyl-1,4-dioxane-2, 5-dione (d,l-lactide), stannous 2-ethylhexanoate, dichloromethane, anhydrous toluene, tetrahydrofuran (THF), Nile red, retinol and 3-(4,5-dimethylthiazol-2-yl)-2,5-diphenyltetrazolium bromide (MTT) were obtained from Sigma-Aldrich (St Quentin-Fallavier, France). Dialysis membrane (molecular weight cut-off (MWCO) 2 kDa, regenerated cellulose) was purchased from BioValley (Marne La Vallée, France). l-Glutathione reduced (GSH), Dulbecco’s modified Eagle medium (DMEM), foetal bovine serum (SVF), dimethyl sulfoxide (DMSO) and penicillin/streptomycin solution were provided by Fisher Scientific (Illkirch, France). Ultrapure water was produced using a Milli-Q system, Millipore (Paris, France).

### 2.2. Synthesis and Characterization of Redox-Responsive (PEG–block–PLA)-Blend-(PLA)

#### 2.2.1. Step 1: Synthesis of Ethanol, 2-[(5-Nitro-2-pyridinyl)dithio]-(Abbreviated as Compound **1**)

In a three-necked flask equipped with a stir bar, under a nitrogen atmosphere and at room temperature, 1.2 g of 2,2′-dithiobis(5-nitropyridine) (310.31 g/mol, 3.87 mmol) were solubilized in 18 mL of dichloromethane. A solution of 2-mercaptoethanol was prepared in 1.5 mL of methanol (275 μL, 78.13 g/mol, 3.87 mmol) and added to the mixture. Finally, 25 μL of anhydrous acetic acid was added. The solution turned yellow and was stirred for 24 h. The solvent was then removed under vacuum and flash chromatography (silica: 60, 0.04–0.063 mm) was carried out using 30% ethyl acetate and 70% cyclohexane to give the compound 1 (232.28 g/mol, 468.5 mg, 2.02 mmol, 52.2% yield) as a yellow solid. ^1^H NMR (300 MHz, CDCl_3_, ppm): δ 9.34 (d, J = 2.1 Hz, 1H), 8.38 (dd, J = 8.8, 2.6 Hz, 1H), 7.69 (d, J = 8.8 Hz, 1H), 3.80 (t, 2H), 3.25 (s, 1H), 3.02 (t, 2H).

#### 2.2.2. Step 2: Synthesis of Disulfide PEG (Abbreviated as **P1**)

In a three-necked flask equipped with a stirring bar, under a nitrogen atmosphere and at room temperature, 500 mg of PEG-thiol (5 kDa, 0.1 mmol), 71.3 mg of compound 1 (232.28 g/mol, 0.3 mmol) and 15 µL of anhydrous acetic acid were solubilized in 23 mL of methanol. The solution turned yellow and was stirred for at least 30 h. The progress of the reaction was followed with a UV-Visible spectrophotometer (Genesys 10S, Thermo Scientific, France) with the release of thionitropyridine in the reaction medium (385 nm). The solvent was then removed under vacuum and the residue was dissolved in distilled water and dialysis was performed against distilled water (regenerated cellulose, MWCO = 2 kDa). Finally, the product was freeze-dried for two days to obtain a white powder stocked at −20 °C. ^1^H NMR (300 MHz, CDCl_3_, ppm): δ 3.87 (t, 2H, J = 4.9 Hz), 3.64 (m, 4H), 3.46 (t, 2H), 3.40 (t, 2H, J = 4.9 Hz), 3.37 (s, 3H), 3.00 (t, 2H, J = 7.0 Hz), 2.87 (t, 2H, J = 5.7 Hz), 2.64 (t, 2H, J = 7.0 Hz).

#### 2.2.3. Step 3: Synthesis of Redox-Responsive Copolymer (Abbreviated as **P2**)

Appropriate amounts of pure d,l-lactide and **P1** were placed in a two-neck flask with anhydrous toluene (4.5 mL) and the mixture was degassed by bubbling nitrogen for 20 min. Under a nitrogen atmosphere, the stannous 2-ethyl-hexanoate (10 mg) was added and the reaction mixture was refluxed in an oil bath (120 °C) for 1 h. During the reaction period, the condenser was cold enough to prevent the volatilization of toluene. The toluene was then removed under reduced pressure and the residue was solubilized in a minimum of tetrahydrofuran (THF) to perform purification by size exclusion chromatography (Sephadex LH-20, THF). The purification of the polymers **P2** produced from the residual lactide monomer was followed by infrared spectroscopy (Bruker Vector 22, ATR mode). After the collection and concentration of polymers under vacuum, it is solubilized in a minimum of THF and precipitated in cold water. After THF removal, freeze-drying was performed for 3 days to obtain a white powder stocked at −20 °C. ^1^H NMR (300 MHz, CDCl_3_, ppm): δ 5.18 (m, 1H), 4.38 (t, 2H, J = 6.6 Hz), 4.35 (q, 1H, J = 7.0 Hz), 3.87 (t, 2H, J = 4.9 Hz), 3.64 (m, 4H), 3.40 (t, 2H, J = 4.9 Hz), 3.37 (s, 3H), 2.97 (t, 2H, J = 7.1 Hz), 2.89 (t, 2H, J = 6.7 Hz), 2.58 (t, 2H, J = 7.2 Hz), 1.57 (m, 3H).

#### 2.2.4. Characterizations

For each step, ^1^H NMR spectra were recorded with a Bruker 300 MHz NMR spectrometer at 25 °C with CDCl_3_ as solvent. MALDI-TOF mass spectra were acquired on a 4700 Proteomics Analyzer (Applied Biosystems). Dithranol (DT; 1,8-dihydroxy-9,10-dihydroanthracen-9-one) was used as the matrix for the ionization. Laser (YAG, 355 nm) power was adapted to obtain a significant signal-to-noise ratio and a good resolution of the mass peaks.

### 2.3. Preparation of Polymer Nanocarriers

Polymer nanocarriers were prepared by the nanoprecipitation method, in triplicate. Briefly, 0.6 mL of a solution of the redox-responsive blends of polymers in acetone (10 mg/mL) was added dropwise to 2 mL deionized water using a syringe pump (KR Analytical, UK) with a flow rate of 0.3 mL/min and under moderate stirring. The nanocarriers were formed instantly, and the organic solvent was evaporated overnight under stirring at room temperature. Blank nanoparticles labelled with Nile red as well as retinol-loaded nanocarriers were prepared by the same method after dissolving Nile red (13 µg/mL) or retinol (1.2 mg/mL), respectively, in the polymer acetone solution. The resulting suspensions were filtered through a 0.45 µm polyether sulfone membrane filter to remove unloaded Nile red or retinol. All the procedures were performed under dark conditions to preserve the integrity of the molecules.

### 2.4. Characterization of Copolymer-Based Nanocarriers

The morphological examination of nanocarriers was conducted by transmission electron microscopy (TEM). The nanocarriers’ aqueous suspension was deposited onto a carbon coated grid and was negatively stained using uranyl acetate (3% *w/v*). Excess water was removed by filter paper and the sample was scanned in a JEOL 1011 TEM (Peabody, MA, USA). 

The mean hydrodynamic diameter (D_H_) and the polydispersity index (PdI) of the nanocarriers in aqueous suspensions were measured by the dynamic light scattering (DLS) technique, using a Nanosizer apparatus (Zetasizer^®^, Malvern Panalytical, UK) equipped with a He-Ne laser (633 nm, scatter angle 173°). Herein, the DLS method measures nanoparticle diameter by intensity. The same instrument was also used for zeta potential measurements, but the samples were put in dedicated micro-electrophoresis cells. Each sample was diluted 1:10 with deionized water and the measurement was performed three times at a temperature of 25 °C.

### 2.5. Redox-Responsivity of Copolymer-Based Nanocarriers

The redox-mediated response to reduced glutathione (GSH) was evaluated by in vitro tests, using blank nanocarriers labelled with environment-sensitive fluorescent dye Nile red (NR-NCs) or retinol-loaded nanocarriers (R-NCs). Typically, the nanocarriers’ suspension was diluted 1:10 with deionized water. Then, 0.5 mL of diluted suspension was mixed with 0.5 mL of GSH solution (0.04 or 20 mM) or 0.5 mL of diluted nitric acid solution to ensure the same pH. Each tube was shaken at 37 °C and Nile red (NR) or retinol (R) fluorescence was measured at predetermined time intervals (0 h, 2 h, 4 h, 8 h, and 24 h) using a spectrofluorometer (A Hitachi F-4500). The fluorescence excitation wavelength was fixed to 535 nm (NR) or 327 nm (R), and the emission fluorescence to 555–700 nm (NR) or 350–650 (R); the range was monitored. Emission and excitation slits were adjusted as function of fluorescence intensity of the solution at t_0_. To study the NR/R release as a function of time after GSH treatment, the following formula was used:

(1) $$\%\;\mathrm{of}\;\mathrm{content}\;\mathrm{released}\;\mathrm{at}\;t=\frac{\mathrm{Fluorescence}\;\mathrm{intensity}\;t_{0}-\mathrm{Fluorescence}\;\mathrm{intensity}\;t}{\mathrm{Fluorescence}\;\mathrm{intensity}\;t_{0}}\times 100$$

The encapsulation efficiency of retinol was measured using its absorbance (A) at 327 nm, after dissolution of the NCs in THF. Appropriate dilution of retinol-loaded nanocarriers’ suspension was performed in THF and the solution was vortexed for 1 min to break down the particles and dissolve retinol. The absorbance of the resulting solution was measured in a 1 cm quartz cell placed in a Genesys 10 S (Thermo Scientific, France) UV-Vis spectrophotometer. From the absorbance values, retinol concentration was determined according to a standard calibration curve within the linear range. The encapsulation efficiency of retinol was calculated according to the following equation:

(2) $$\mathrm{Encapsulation}\;\mathrm{Efficiency}\;(\%)=\frac{\mathrm{Amount}\;\mathrm{of}\;\mathrm{retinol}\;\mathrm{in}\;\mathrm{the}\;\mathrm{nanocarriers}}{\mathrm{Feeding}\;\mathrm{amount}\;\mathrm{of}\;\mathrm{retinol}}\times 100$$

The theoretical loading was calculated by Equation (3):

(3) $$\mathrm{Molecule}\;\mathrm{load}\;(\%)=\frac{\mathrm{Determined}\;\mathrm{weight}\;\mathrm{of}\;\mathrm{retinol}}{\mathrm{Initial}\;\mathrm{weight}\;\mathrm{of}\;\mathrm{copolymer}}\times 100$$

Aqueous suspensions are divided into two samples and transferred into glass vials immediately after preparation and stored at 4 °C or room temperature. The chemical stability was monitored up to 1 month by drug content determination. Periodically, samples were taken and diluted with THF and analyzed with the UV-Vis spectrophotometer for residual retinol.

### 2.6. In Vitro Cytotoxicity Studies of Retinol-Loaded Nanocarriers

In vitro cytotoxicity of blank and retinol-loaded nanocarriers was evaluated by the 3-(4,5-dimethylthiazol-2-yl)-2,5-diphenyl-tetrazolium bromide (MTT) assay on the HaCaT cell line (human keratinocytes, a kind gift from Pr. G. Weber, U1069 N2C, Tours) [41]. Cells were cultured in DMEM completed with 10% of SVF and 1% of a penicillin–streptomycin mixture. For the cytotoxicity study, cells were seeded in 96-well plates (10,000 cells/well) and incubated 24 h at 37 °C in a humidified atmosphere with 5% CO_2_. Cells were then treated with increasing concentrations of blank or retinol-loaded nanocarriers (1.64 × 10^−7^ to 1.64 × 10^−2^ mg/mL in retinol) during 24 h at 37 °C. MTT solution was added to each well (0.5 mg/mL) and the cells were incubated for further 4 h at 37 °C. Then, the supernatant was discarded and DMSO was added in each well to dissolve the formazan crystals. The absorbance A at 540 nm was measured with a microplate reader (Bio-tek EL800 plate reader). Results were expressed as viability percentages as function of the retinol concentration, and compared with Student’s *t*-test.

## 3. Results and Discussion

### 3.1. Synthesis and Characterization of Redox-Responsive PEG–block–PLA

**Figure 1:**
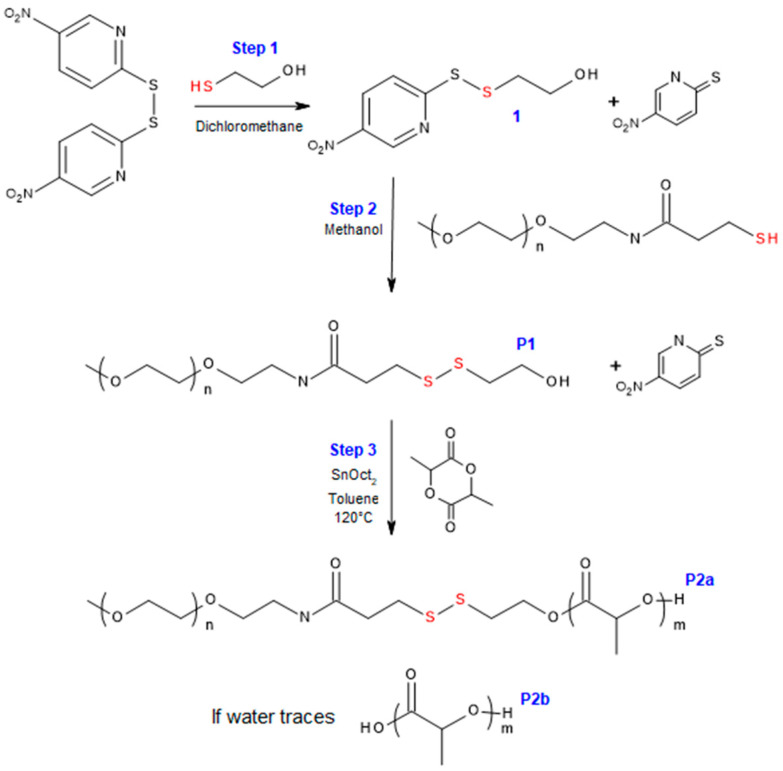
Synthesis of the amphiphilic redox-responsive poly(ethylene glycol)–*block*–poly(lactide) (PEG–*block*–PLA) in three steps.

The redox-responsive PEG–*block*–PLA was synthesized in three steps, as described in Figure 1.

Unable to avoid the presence of traces of water after purification of **P1**, two compounds are expected at the end of step 3: redox-responsive PEG–*block*–PLA (**P2a**) and PLA (**P2b**).

**Figure 2:**
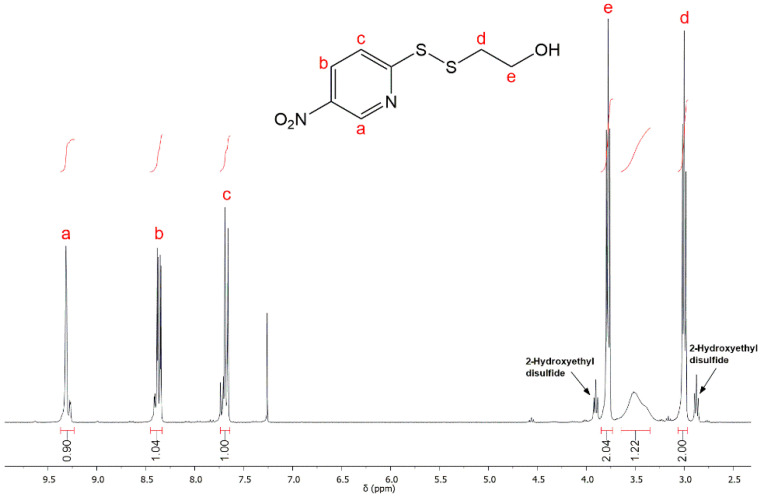
^1^H NMR spectrum of compound 1 (step 1, CDCl_3_, 25 °C).

The first step consists of producing an intermediate product, called compound 1, with a disulfide linkage. Its composition and structure were confirmed by ^1^H NMR as shown in Figure 2.

The solvent peak of CDCl_3_ was found at 7.26 ppm. Peaks at 2.87 and 3.90 ppm result from the oxidation of 2-mercaptoethanol to 2-Hydroethyldisulfide. Peaks at 3.8 and 3.02 ppm were assignable to the methylene (–CH_2_–) protons of –CH_2_–OH (e) and –S–CH_2_– (d), respectively.

**Figure 3:**
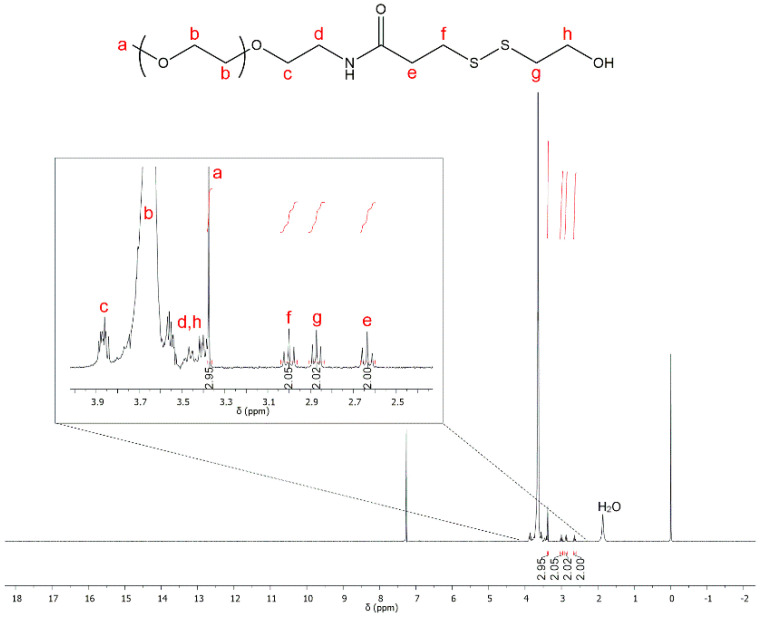
^1^H NMR spectrum of the modified polymer **P1** (step 2, CDCl_3_, 25 °C).

The second step consists of thiol–disulfide exchange between compound 1 and PEG-thiol in order to obtain the polymer **P1**. The reaction yield was established by following the release of thionitropyridine from compound 1 in the reaction medium with a UV-Vis spectrophotometer (λ_max_ = 385 nm). After approximately 30 h of reaction, the yield was close to 100%. At this stage, the elimination of the remaining quantity of compound 1 is crucial because the next step is the polymerization of PLA, possible from a hydroxyl group. To purify the hydrophilic polymer **P1**, a common laboratory technique was used, the dialysis method. This method is vulnerable to the emergence of traces of water in the reaction medium that would initiate the synthesis of PLA. The composition and the structure of the purified **P1** product were investigated with NMR and MALDI-TOF mass spectrometry. Figure 3 shows ^1^H NMR spectrum of the modified PEG produced.

In NMR investigation, the signal at 3.68 ppm was assigned to the methylene protons of PEG units (–O–CH_2_–, b). The signal at 3.37 ppm can be assigned to the three chemically equivalent hydrogen atoms of the terminal methoxy group (–OCH_3_, a) of the mPEG *block*. The triplet peaks at 3.87, 3.40, 3.00 and 2.64 ppm were attributed to the methylene protons (–CH_2_–) of the PEG_5K_-SH (c, d, f and e, respectively). Finally, peaks at 3.46 and 2.87 ppm were assignable to the insertion of the methylene (–CH_2_–) protons of compound 1: –CH_2_–OH (h) and –S–CH_2_– (g), respectively. As expected, water traces are observed.

**Figure 4:**
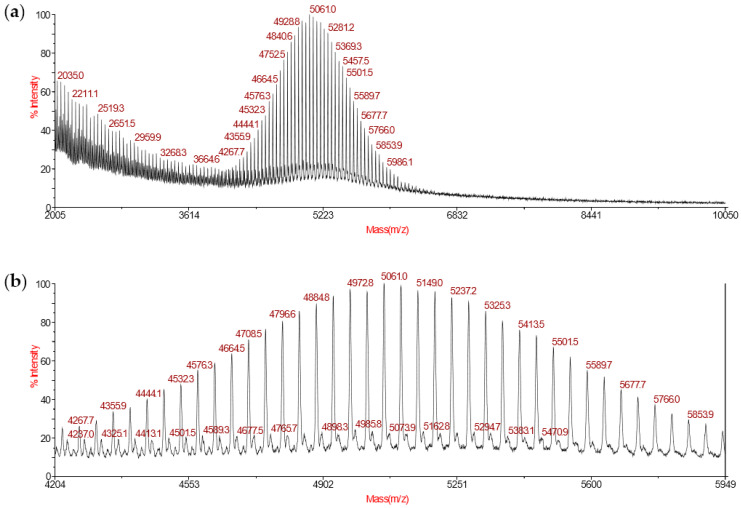
(**a**) Full MALDI-TOF mass spectrum from mass/charge (m/z) 2000 to 10,050 of **P1**. (**b**) Magnification of the mass range region from m/z 4204 to 5949.

Mass spectrometry was used as a complementary method to conclude if the disulfide linkage was intact. Figure 4 shows the MALDI-TOF mass spectrum of the purified product of step 2.

The MALDI-TOF mass spectrum was well-resolved and the peaks were separated by 44 mass units, which corresponds to the molecular weight of the ethylene oxide (EO) unit of PEG. Two populations were observed in the mass range region from m/z 4204 to 5949 (Figure 4b). The major population corresponds to the modified PEG **P1** and the minor population corresponds to the unmodified mPEG-thiol, which has not reacted with compound 1. Thanks to mass spectrometry analysis, the integrity of the disulfide linkage of **P1** was confirmed.

**Figure 5:**
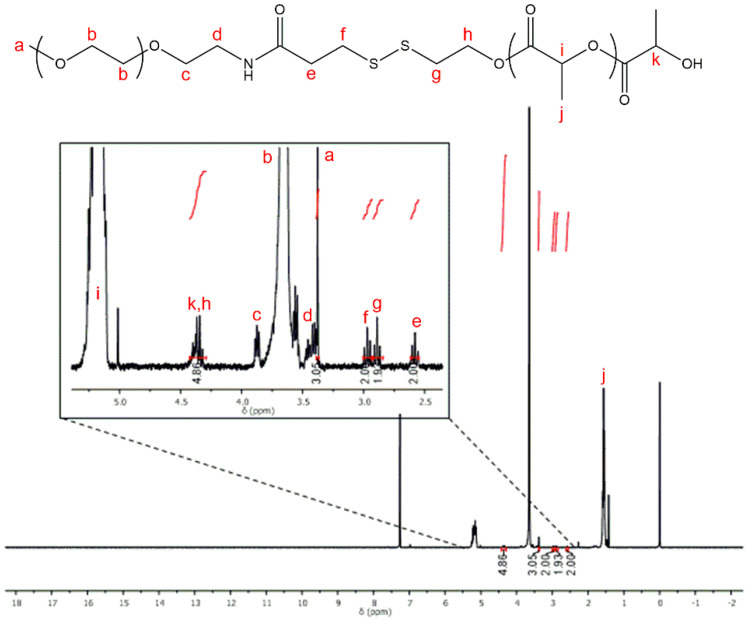
^1^H NMR spectrum of **P2** copolymer (step 3, CDCl_3_, 25 °C).

The last step consists in producing the copolymer PEG–*block*–PLA (**P2a**) by ring-opening polymerization (ROP). The hydroxyl functional group at the end of the modified PEG **P1** allows the ROP of d,l-lactide using stannous octoate (SnOct_2_) as the catalyst [6,7,42]. Even after freeze-drying of the modified PEG, traces of water persist and are susceptible to participate in the polymerization of PLA (**P2b**). The copolymer composition was confirmed by ^1^H NMR as shown in Figure 5. The peaks at 1.57 and 5.18 ppm belong to methyl (–CH_3_, j) and methine (–CH, i) protons of the lactate unit of PLA, respectively. The signal at 3.68 ppm (b) was assigned to the methylene protons of PEG units. The methylene protons signal (–CH_2_–, h) of **P1**, linked to the hydroxyl group, shifts from 3.46 to 4.4 ppm after polymerization. This signal is superimposed with that corresponding to the PLA end-group methide proton (–CH, k). If the final product corresponded to only **P2a** copolymer, the integration at 4.4 ppm was then supposed to be three (=3H). Herein, the integration was 4.86 corresponding to 2H of methylene protons (–CH_2_–, h) and 2.86H associated to PLA end-group methide protons. The assumption is as follows: among the 2.86 protons, 1H corresponds to the PLA end-group methide proton of **P2a** and 1.86H corresponds to the PLA end-group methide protons of PLA polymerized with water traces (**P2b**). The NMR investigation could be interpreted as follows: we have a blend of copolymer **P2a** and polymer **P2b** with an approximate ratio of 1:2. According to the initial amount of polymer **P1** involved in the reaction (~200 mg), the amount of water traces was evaluated as ~8.3 × 10^−5^ moles.

**Figure 6:**
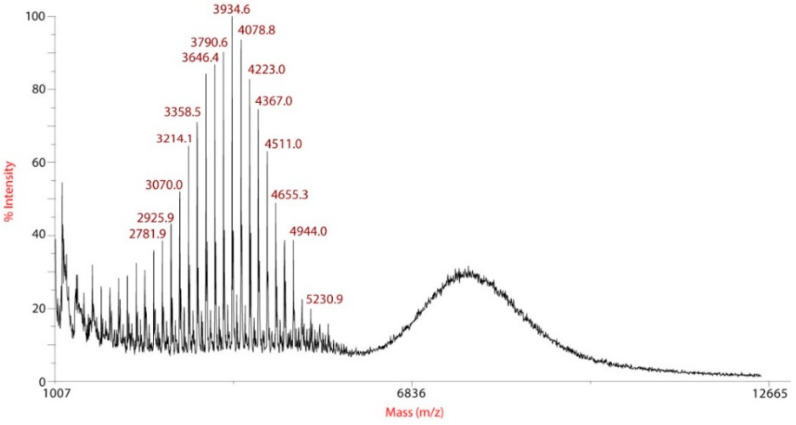
MALDI-TOF mass spectrum of purified product of step 3 (m/z 1007 to 12,665).

Usually, integrations of PEG and PLA protons are used to determine the molecular weight of the copolymer. Being in the presence of a blend, a MALDI-TOF mass spectrum was recorded as a complementary method to properly study the composition of the final product (Figure 6). The population below 5.5 kDa corresponds to the PLA polymer with an increment of 144 m/z corresponding to the lactate unit of PLA. This population is associated to the PLA polymerized from water traces with a molecular weight Mn_PLA_ = 3637 Da (PLA_4K_). The population observed above 5.5 kDa corresponds to the copolymer PEG–*block*–PLA **P2a** with a molecular weight close to 8 kDa corresponding to a PEG of approximately 5 kDa and a PLA of approximately 3 kDa.

To conclude, thanks to NMR and mass investigations, the blend of polymers was characterized, and it is composed of the copolymer PEG–*block*–PLA (**P2a**) and the polymer PLA (**P2b**) with an approximate ratio 1:2. No further purification was performed to explore directly the interest of the mixture for nanocarrier development, minimizing steps for industrial manufacturing. This polymer will be referred to (PEG–*block*–PLA)-blend-(PLA) in the rest of the document.

### 3.2. Nanocarriers Characteristics

The nanoprecipitation method was used to produce (PEG–*block*–PLA)-blend-(PLA) nanocarriers [43]. This method allows a spontaneous particle formation with no surfactant and with low energy consumption [40,44]. The miscibility of PLA and PEG, added to the fact that both polymers possess a PLA chain, should ensure the coprecipitation of the polymer in mixed nanospheres [45]. The nanocarriers prepared from (PEG–*block*–PLA)-blend-(PLA) were stable in aqueous suspension without the addition of surfactant. This stability is in favor of a core-shell structure where the PLA blocks make up the inner core while the majority of the hydrophilic PEG blocks make up the outer hydrophilic shell [22,40]. Nevertheless, the nanoprecipitation method, compared to the emulsion/solvent evaporation or emulsion/solvent diffusion used to prepare nanosystems, may lead to less organized systems. Blank nanoparticles were labeled with Nile red (~0.1% *w/w*) in order to be able to follow their behavior in a reducing medium.

**Figure 7:**
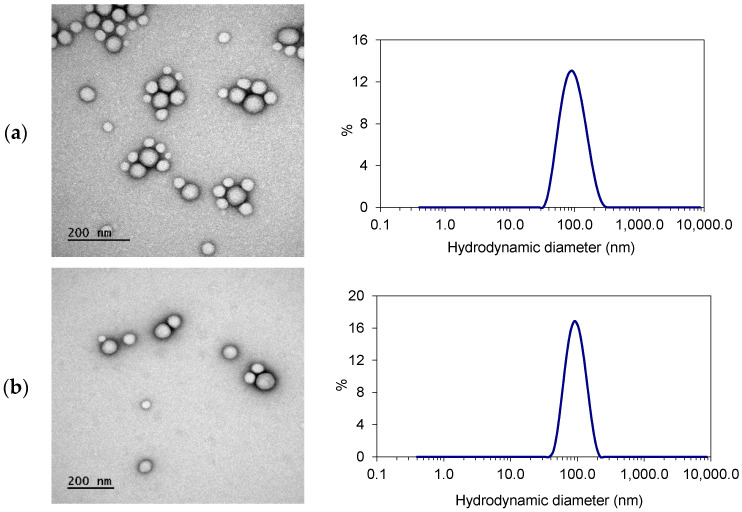
Typical TEM images and representative particle size distribution plots of (**a**) Nile red-labelled and (**b**) retinol-loaded (PEG–*block*–PLA)-blend-(PLA) nanocarriers.

As can be seen in TEM images (Figure 7), Nile red labelled and retinol-loaded nanocarriers (NR-NCs and R-NCs, respectively) have similar spherical morphologies and sizes. The particle size distribution shows only one population of nanocarriers with narrow distribution, suggesting a monodispersed population of particles. This monodispersity is in line with the hypothesis that both polymers coprecipitate to form composite nanospheres and not two separate populations: one made of redox sensitive PEG-*block*-PLA and one made of PLA.

The physicochemical characteristics of the labelled and retinol-loaded nanocarriers are compiled in Table 1. Depending on the copolymer composition, method and experimental conditions of formulation, the size of the PEG–*block*–PLA nanocarriers described in the literature may vary from 50 to 300 nm [46]. In this study, the hydrodynamic diameter of blank nanocarriers labelled with Nile red or loaded with retinol was close to 100 nm, which can be considered suitable for skin administration [47]. As expected, D_H_ values obtained from DLS measurements were higher than NP diameters observed in TEM images: the former includes hydrated layers of PEG shell, while the latter may not reveal PEG, because of its low electronic contrast.

NCs’ aqueous suspensions exhibit low PdI values (<0.2) indicative of populations of rather narrow size distribution. They show high negative zeta potentials (−22 to −28 mV), similar to those of PEG–*block*–PLA-based nanosystems already described [48,49]. These surface charges suggest that electrostatic repulsion of NCs should significantly favor their colloidal stability and reinforce PEG steric hindrance.

As the particle size is linked to the size of the droplets generated during the nanoprecipitation, it is not surprising that blank and retinol-loaded NCs show similar sizes. Nevertheless, their internal structure is in all likelihood different. Indeed, R-NCs show a high loading of ~9.2% *w/w* of retinol. The introduction of nearly 10% of hydrophobic chains could lead to a different stability or release kinetics of blank and loaded NCs. The chosen length of the PLA chains, deliberately short, led to an effective retinol encapsulation. The encapsulation efficiency (EE) of retinol was of 76.9 ± 11.6% *w/w*, which is satisfactory compared to the literature data on retinol nanocarriers such as (PEG–*block*–PLA)-based NCs (PLA of ≈15 kDa, EE = 100% [34]), chitosan nanocarriers (EE = 76%, [32]), silicone and silica particles (EE = 85% [31] and 31% [33], respectively), and solid lipid nanocarriers (EE = 74% or up to 97% depending on formulation conditions [50,51]). The nanoprecipitation method and experimental conditions described in this study allow a high encapsulation efficiency of retinol into (PEG–*block*–PLA)-blend-(PLA) nanocarriers, with a final retinol concentration in water of 28% *w/v* (Table 1).

**Figure 8:**
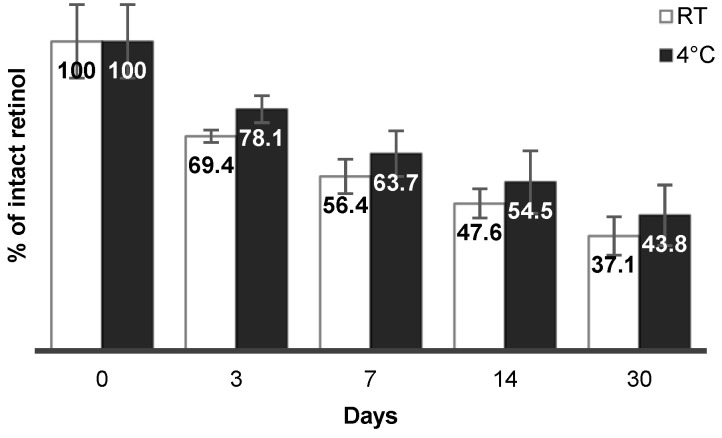
Chemical stability at room temperature (white) and 4 °C (black) of retinol incorporated into nanocarriers.

The size, PdI and zeta potential of the nanocarriers do not vary significantly over one month, showing the physical stability of such a suspension of nanocarriers. Since retinol is known to be chemically unstable, the stability of retinol is one of the most important factors during storage [27,28,29]. Retinol-loaded nanocarrier suspensions were stored in darkness at room temperature (RT) or 4 °C for 1 month. The chemical stability of retinol is reported in Figure 8.

Stabilizers are usually required to improve the long-term stability of retinol, such as butyl hydroxy toluene (BHT), an antioxidant which protects retinol from chemical degradation [52]. Herein, no stabilizer was added and, after one month in darkness at room temperature or 4 °C, there remained ~40% of non-degraded retinol. These results are encouraging compared to the literature. Indeed, several formulations of retinol have been developed with variable chemical stabilities specific to nanocarriers and development conditions. For example, retinol was encapsulated with BHT in silicone particles for topical delivery and after 14 days in darkness at 45 °C, there remained ~25% of non-degraded retinol [31]. Retinol was also encapsulated in silica particles where the amount of preserved retinol depended on the concentration of surfactants and PEG polymer. With 13 and 1.3% *w/w* of Span^®^80 and Tween^®^80, respectively, there remained ~85% of intact retinol after 6 days [33]. Solid lipid nanocarriers (SLNs) showed almost 100% of residual retinol after 4 weeks, at room temperature (shade) or 4 °C [50]. In another study, 43% of retinol remained intact in SLNs after 12 h at room temperature (shade). However, the instability of retinol could be overcome by co-loading of antioxidants such as BHT in SLNs [51]. Furthermore, the protection of retinol in R-NCs could be enhanced by the inclusion of stabilizers like BHT or retinol-palmitate in the organic phase before nanoprecipitation.

### 3.3. Nanocarriers Behaviour in a Reductive Medium

**Figure 9:**
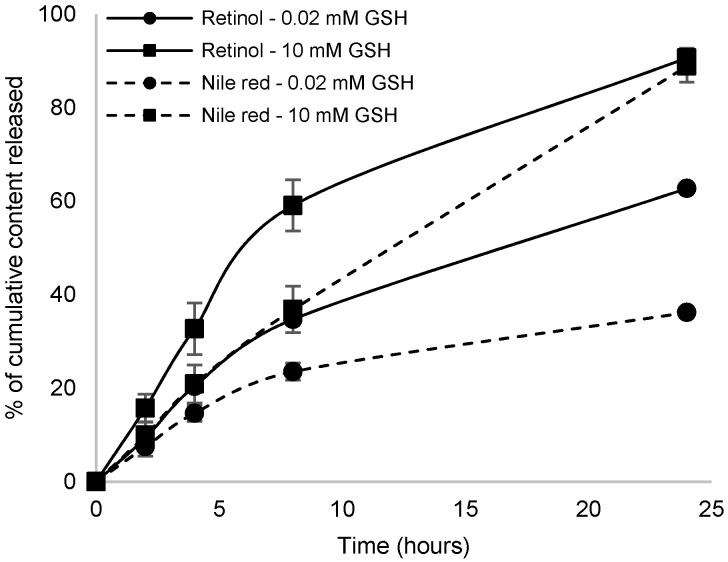
In vitro release of Nile red (dashes) and retinol (full line) from (PEG–*block*–PLA)-blend-(PLA) nanocarriers at 37 °C with glutathione (GSH basal level (0.02 mM) and GSH activation level (10 mM).

Glutathione (GSH) is a physiological detoxification molecule showing a highly reactive thiol function. It has been reported that, in the cytosol and nuclei, the concentration of GSH can reach 10 mM, while outside the cell the concentration is about 2–20 µM [53,54,55]. The insertion of the disulfide linkage into the copolymer-based nanocarriers is supposed to accelerate the release of the cargo in a GSH-rich environment [6]. Nile red-labelled blank NCs and retinol loaded NCs were used to investigate their redox response in vitro. Fluorescence spectra (λ_ex_ = 535 nm for NR and 327 nm for retinol) were recorded from diluted aqueous suspensions, exposed or not to GSH treatment, at different incubation times, at 37 °C. NR fluorescence is strongly influenced by its molecular environment. If NR is released from the hydrophobic environment of nanocarriers in the surrounding hydrophilic environment, its fluorescence yield dramatically decreases [56]. In addition, when retinol is released from the hydrophobic environment of nanocarriers, it is very quickly degraded and loses its fluorescence properties in the measurement conditions described in the Materials and Methods section. Indeed, the UV-visible spectrum of free retinol displays a band shift from 327 nm to 282 nm after a few minutes in water, revealing the disappearance of highly conjugated structures (See Figure S1 in Supplementary Materials). This phenomenon is not disrupted by the presence of GSH. It allowed us to track, by fluorescence, the relative amount of retinol still encapsulated. The NR or R fraction released from (PEG–*block*–PLA)-blend-(PLA) was estimated as described in the Experimental section and release kinetics curves were established (Figure 9). These curves show a spontaneous release of encapsulated molecules from the particles with a basal concentration of GSH, but this release is accelerated by the presence of high a concentration of GSH. During the first 2 h of incubation, the NR or R release was similar to the GSH basal level or with GSH activation level. The release seems thus independent of the response to a stimulus over the first 2 h. It is supposed that fluorescent molecules present near the surface of the nanocarriers might be released at early stages. Nevertheless, this release is progressive and cannot be qualified as burst release. From 4 h of incubation, the cumulative release of NR or retinol was higher in GSH-enriched environment compared to the control. This difference becomes even more significant after 24 h: 89 ± 3% vs. 36 ± 2% for Nile red and 91 ± 2% vs. 63 ± 0.1% for retinol. Retinol is released faster than Nile red, which could be explained by the difference in initial content and in NCs’ internal structures. Indeed, retinol being more widely present in nanocarriers, their polymeric structure may be degraded more rapidly or the structure more easily destabilized than blank nanoparticles comprised of 99% polymer.

The release profile of retinol from (PEG-*block*-PLA)-based nanocarriers has not yet been reported in the literature, certainly because of retinol instability. However, (PEG–*block*–PLA)-based nanocarriers have been described for the protection and release of all-*trans* retinoic acid (atRA, log P ~ 5) [48,49]. The atRA cumulative release depends on hydrophobic and hydrophilic chain lengths. In Tiwari et al.’s study, atRA-loaded (PEG–*block*–PLA)-based nanocarriers produced by the nanoprecipitation method reached a size close to 150 nm. The atRA cumulative release reached ~7.5% after 24 h [49]. In fact, the strategy described in this paper could be applied to retinoids in general to achieve an accelerated release. Even if those studies are not directly comparable, the GSH-enhanced release of hydrophobic content is consistent with the supposed mechanism of the NC degradation via disulfide bonds cleavage [57,58]. The more plausible explanation according to the literature is that the PEG hydrophilic chains are then detached from the hydrophobic PLA core causing the destabilization of the NCs leading to the higher release of the contents [6]. This destabilization is even more efficient if the structure of the nanocarriers includes residual PEG chains in the core of the NCs. TEM images of R-NCs degraded after GSH treatment are shown as supplementary information (Figure S2 in Supplementary Materials) and are consistent with this hypothesis. Those results show that a ratio 1:2 between (PEG–*block*–PLA) and PLA permits to prepare stimuli-responsive nanosystems.

### 3.4. In Vitro Cytotoxicity Studies of Retinol-Loaded Nanocarriers

**Figure 10:**
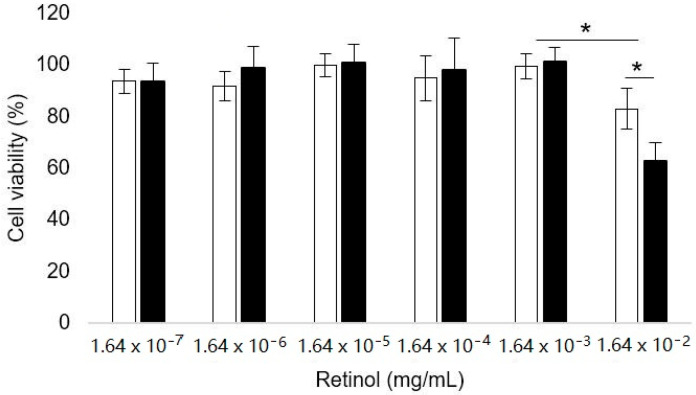
Cell viability of human keratinocytes (HaCaT) exposed to increasing concentrations of blank nanocarriers (open bars) and retinol-loaded nanocarriers (filled bars). Mean ± SD, *n* = 6. * *p* < 0.05.

Cytotoxicity of (PEG–*block*–PLA)-blend-(PLA) nanocarriers towards skin cells is a key index for their topical use. The results on cell viability of human keratinocytes (HaCaT cells) exposed to increasing concentrations of blank and R-NCs (see Figure 10).

According to the literature, blank and retinol-loaded nanocarriers suspensions can be considered non-toxic to human keratinocytes up to a corresponding retinol concentration of 1.64 × 10^−3^ mg/mL (viability > 70%) [59]. At higher retinol concentrations (1.64 × 10^−2^ mg/mL), human keratinocyte viability decreased to 83% for blank nanocarriers and 63% for retinol-loaded nanocarriers. It is not surprising because retinol is often described as toxic depending on the concentration, generating skin irritation [31].

To conclude, retinol-loaded (PEG–*block*–PLA)-blend-(PLA) nanocarriers show a satisfactory biocompatibility for their skin application. Nonetheless, the toxicity can be increased by the encapsulation of active molecules with intrinsic toxicities.

## 4. Conclusions

Thanks to the simplified synthesis described in this study, it was possible to prepare a redox-responsive blend of PEG–*block*–PLA and PLA with a 1:2 ratio. Smart nanocarriers were formulated by the nanoprecipitation method. Retinol, a hydrophobic and unstable anti-ageing agent, was used as a model active molecule. The strategy to deliberately choose short-length polymers to encapsulate retinol permitted the reaching of high loading and rapid release in a reducing medium in vitro. Nevertheless, the co-encapsulation of stabilizers could increase the chemical stability of the system. Blank NCs showed a good biocompatibility with respect to human keratinocytes, but (PEG–*block*–PLA)-blend-(PLA) NCs can become toxic as function of the concentration of encapsulated molecule, depending on the toxicity of the molecule itself. These nanocarriers could be used to deliver other retinoids for treatment of skin diseases like acne, photoageing, psoriasis vulgaris, melisma or skin cancers [60,61]. In a more general manner, such redox-responsive nanocarriers could have a high interest for biologically controlled delivery of hydrophobic active molecules in dermatological or cosmetic applications. This study shows that it is not necessary to work in drastic anhydrous conditions and to purify the redox-responsive polymer to obtain a triggered release of disulfide-linked (PEG–*block*–PLA)-blend-(PLA)-based nanocarriers. This simplified copolymer synthesis coupled to nanoprecipitation offers perspective to the industrialization of stimuli-responsive nanosystems.

## Figures and Tables

**Table 1:** Characteristics of nanocarriers (mean values ± SD, n = 3).

	Characteristics	Nile Red-Labelled Nanocarriers	Retinol-Loaded Nanocarriers
Day 1	Hydrodynamic diameter (nm)	100.5 ± 2.7	98.7 ± 2.2
	Polydispersity Index	0.126 ± 0.013	0.101 ± 0.011
	Zeta potential (mV)	−25.7 ± 2.3	−21.6 ± 1.6
	Retinol (mg/mL)	-	0.28 ± 0.04
1 month 4 °C	Hydrodynamic diameter (nm)	98.0 ± 2.4	94.1 ± 1.6
	Polydispersity Index	0.115 ± 0.017	0.093 ± 0.010
	Zeta potential (mV)	−27.8 ± 3.1	−20.7 ± 3.6
	Retinol (mg/mL)	-	0.12 ± 0.01

## Supplementary Materials

The following are available online at https://www.mdpi.com/2073-4360/12/10/2350/s1, Figure S1: Absorbance spectra of retinol-loaded copolymer-based nanoparticles (black); retinol in THF (violet), retinol in distilled water (blue, full line) and retinol in distilled water containing glutathione, 10 mM (blue, dashes); Figure S2. Typical TEM images of retinol-loaded nanocarriers incubated 24 h at 37 °C (a) without and (b) with GSH treatment (10 mM).
