# Supplementary material for: Three-Step Synthesis of a Redox-Responsive Blend of PEG–block–PLA and PLA and Application to the Nanoencapsulation of Retinol
Source: Polymers (Basel). 2020 Oct 14;12(10):2350. doi: 10.3390/polym12102350 (PMC7602167; doi:10.3390/polym12102350)
Supplement: Supplementary file 1 [file polymers-12-02350-s001.pdf]

polymers

Supplementary information

S1: Degradation of retinol released in water observed by UV-vis spectroscopy

UV-visible spectroscopy permits to observe retinol degradation in water. When encapsulated in the hydrophobic core of the nanocarriers, retinol spectrum in water is very close to its spectrum in ethanol. When it is dispersed in water, the maximum of absorption at 327 nm disappears quickly. The presence of gluthatione leads to light scaterring.

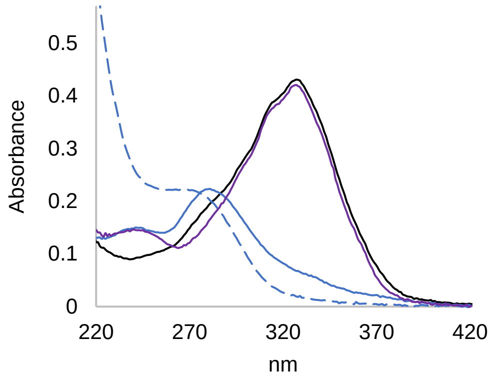

Figure 1. Absorbance spectra of retinol-loaded copolymer-based nanoparticles (**black**); retinol in THF (**violet**), retinol in distilled water (**blue**, full line) and retinol in distilled water containing glutathione, 10 mM (**blue**, dashes).

S2: Impact of glutathione on redox sensitive PEG–*block*–PLA and PLA nanocarriers

TEM images of retinol-loaded nanocarriers, treated or not with GSH (after 24h at 37 °C), were performed. The disruption of the disulfide bond by GSH is supposed lead to the separation of PEG and PLA. PEG are not visible on TEM images because it is transparent to electrons, but after GSH treatment, nanocarriers are still observed, although smaller and less spherical, which can be interpreted as a degradation. Moreover, released retinol could form aggregates, visible in TEM images. The destabilization of nanocarriers by GSH, at the origin of the acceleration of the release of retinol, cannot be proven only by TEM images.

DLS measurements were also done after 24 h of treatment with 10 mM of GSH: the sample is still monodisperse with a hydrodynamic diameter decreased of nearly 10% for both retinol and Nile red. These observation could be consistent with a destabilization of the nanocarriers by the disruption of the disulphide bond.

(**a**) (**b**)

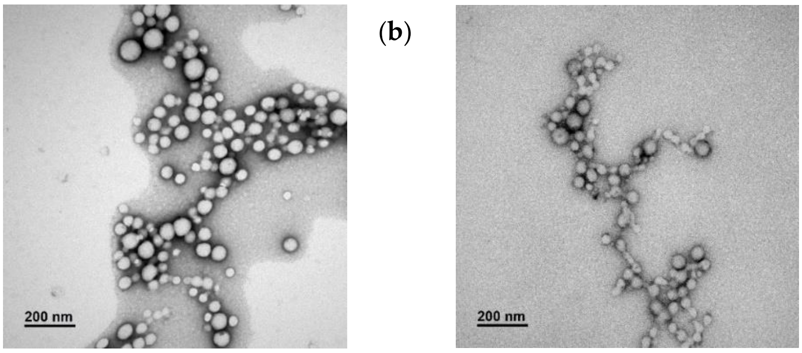

Figure 2. Typical TEM images of retinol-loaded nanocarriers incubated 24h at 37 °C (**a**) without and (**b**) with GSH treatment (10 mM).
